# Rostral cranial fossa as a site for cerebrospinal fluid drainage – volumetric studies in dog breeds of different size and morphotype

**DOI:** 10.1186/s12917-018-1483-3

**Published:** 2018-05-18

**Authors:** Wojciech Sokołowski, Norbert Czubaj, Michał Skibniewski, Karolina Barszcz, Marta Kupczyńska, Wojciech Kinda, Zdzisław Kiełbowicz

**Affiliations:** 1 Department of Morphological Sciences, Faculty of Veterinary Medicine, Warsaw University of Life Sciences – SGGW, Nowoursynowska 159, 02-776 Warsaw, Poland; 2 Department of Surgery, Faculty of Veterinary Medicine, Wroclaw University of Environmental and Life Sciences, Pl. Grunwaldzki 51, 50-366, Wroclaw, Poland

**Keywords:** Hydrocephalus, Lymphatic drainage, Cerebrospinal fluid, Dog, Rostral cranial fossa, Cribriform plate

## Abstract

**Background:**

Hydrocephalus is a multifactorial condition, whose aetiology is not fully understood. Congenital hydrocephalus frequently occurs in small and brachycephalic dog breeds. Although it is widely accepted that the cribriform plate located in the rostral cranial fossa (RCF) is a site of cerebrospinal fluid (CSF) drainage, the RCF has not been studied extensively. Literature reports indicate that a decreased caudal cranial fossa (CCF) volume in the course of the Chiari-like malformation may obstruct CSF circulation. We hypothesised that morphological diversity among different breeds in the volume of the RCF may affect CSF circulation. The aim of the study was to carry out a volumetric analysis of the RCF and the cranial cavity and to determine the ratio between them in dog breeds of different size and morphotype. We performed computed tomography (CT) morphometric analysis of the RCF compartment by obtaining volume measurements from the transverse and reformatted sagittal and dorsal planes.

**Results:**

The rostral cranial fossa percentage – volume of the rostral cranial fossa/volume of cranial cavity × 100 (volRCF/volCC × 100) was lower in small and brachycephalic dog breeds than in the other dogs.

**Conclusions:**

A reduced RCF volume was detected in small and brachycephalic dog breeds, some of which are predisposed to congenital hydrocephalus. This may lead to overcrowding of brain parenchyma in the RCF and may impede CSF circulation. Our observations may be useful for future studies focusing on the causes and new therapies to treat conditions such as hydrocephalus and syringomyelia.

**Electronic supplementary material:**

The online version of this article (10.1186/s12917-018-1483-3) contains supplementary material, which is available to authorized users.

## Background

Hydrocephalus is a pathological condition caused by an imbalance between cerebrospinal fluid (CSF) production, flow and drainage. This results in an accumulation of CSF in the intracranial space, most commonly within the ventricular system [1]. Hydrocephalus is multifactorial condition. Small and brachycephalic breeds are predisposed to congenital hydrocephalus [2, 3]. The arachnoid villi, located in venous sinuses, and nasal lymphatics (NL) are the main site of CSF absorption. CSF enters to the nasal cavity and NL through the cribriform plate located in the rostral cranial fossa (RCF) [1, 4, 5]. Studies suggest that CSF is mainly absorbed into the NL if the intracranial pressure is normal. A disruption of this process may contribute to the development of central nervous system disorders, including hydrocephalus [6–8].

As opposed to the caudal cranial fossa (CCF), the RCF has not been studied volumetrically in dogs. The CCF structures have been studied extensively due to the occurrence of the Chiari-like malformation (CM) in some breeds of small dogs [9–12]. CM has been frequently diagnosed in the Cavalier King Charles Spaniel and is associated with a disproportion between the volume of brain parenchyma and the CCF volume. The reduced CCF volume causes overcrowding of the cerebellum, pons and medulla and may cause syringomyelia (SM) due to disturbances in CSF circulation [13, 14].

We assumed that similar relationships may occur in the RCF. We hypothesised that in small and brachycephalic breeds predisposed to hydrocephalus the volume ratio of the RCF to the cranial cavity (CC) is smaller than in the others. That may potentially cause “overcrowding” of brain structures and disrupt CSF flow through the cribriform plate to the NL. The aim of the study was to carry out a volumetric measurement of the RCF and CC and to assess the relationship between those parameters in dog breeds of various size and morphotype.

## Results

### Morphotype

Individuals were divided into four groups based on skull index. Dolichocephalic breed group (skull index ≤50.00) consisted of 13 individuals (7 males and 6 females). Mesaticephalic 1 breed group (skull index 50.01–65.00) contained 33 individuals (16 males and 17 females). There were 12 animals in mesaticephalic 2 breed group (skull index 65.01–80.00) (9 males and 3 females). Brachycephalic breed group (skull index ≥80.01) comprised 13 animals (3 males and 10 females). The different dog breeds in each group are presented in Table 1. Values of the rostral cranial fossa percentage were lower in group B than in the remaining groups (Table 2). There were statistically significant differences in the analysed parameters between brachycephalic and dolichocephalic breed group (*p* = 0.0002) and brachycephalic and mesaticephalic 1 breed group (*p* = 0.0024). There were no statistically significant differences among the remaining groups.

**Table 1:** The number of dogs of different breeds in each group, division based on the skull index

Dolichocephalic		Mesaticephalic 1		Mesaticephalic 2		Brachycephalic	
Breed	Number of dogs	Breed	Number of dogs	Breed	Number of dogs	Breed	Number of dogs
Black Russian Terrier	1	American Staffordshire Terrier	4	American Staffordshire Terrier	4	English Bulldog	2
Dobermann Pinscher	1	Australian Shepherd	1	Boxer	3	French Bulldog	6
German Shepherd	6	Bavarian Mountain Hound	1	Cavalier King Charles Spaniel	1	Pekingese	5
Great Dane	1	Bernese Mountain Dog	1	Italian Mastiff	1		
Newfoundland	1	Bull Terrier	1	Miniature Pinscher	1		
Scottish Terrier	1	Dachshund	3	Rottweiler	1		
White Swiss Shepherd	1	Dalmatian	2	Yorkshire Terrier	1		
Wire Fox Terrier	1	English Beagle	1				
		German Shepherd	3				
		German Wirehaired Pointer	1				
		Italian Mastiff	1				
		Labrador Retriever	3				
		Miniature Pinscher	2				
		Rottweiler	3				
		Samoyed	1				
		Schipperke	1				
		Standard Schnauzer	1				
		Yorkshire Terrier	3

**Table 2:** Statistical parameters for the rostral cranial fossa percentage for each group

Group	Arithmetic Mean	Range	Median	Standard Deviation	Lower Quartile	Upper Quartile
All Dogs	9.803	6.293–15.194	9.857	1.426	8.671	10.718
Division based on the skull index						
Dolichocephalics	10.989	9.922–12.257	11.057	0.651	10.534	11.257
Mesaticephalics 1	9.970	7307–15,194	10.214	1.502	8.864	10.718
Mesaticephalics 2	9.727	8.135–11,038	9.585	0.851	9.230	10.421
Brachycephalics	8.261	6.293–8985	8.449	0.758	7.999	8.874
Division based on the breed size						
Small Breeds	8.511	6.293–11.114	8.568	0.957	8.096	8.864
Medium Breeds	10.091	8.877–12.007	9.857	0.850	9.723	10.505
Large Breeds	10.661	7.908–12.257	10.728	0.924	10.305	11.062
Giant Breeds	10.939	9.488–15.194	10.499	1.699	10.214	11.153

### Breed size

Individuals were divided into four groups based on the breed size. Small breed group contained 25 individuals (10 males and 15 females). Medium breed group comprised 17 individuals (9 males and 8 females). There were 20 animals (7 males and 13 females) in large breed group and nine in giant breed group (3 males and 6 females). The number of dogs of each breed is presented in Table 3. The rostral cranial fossa percentage reached lower values in small breed group than in the remaining groups (Table 2). There was a statistically significant difference between small and medium breed group (*p* = 0.0002), small and large breed group (*p* = 0.0002) and small and giant breed group (*p* = 0.0002). There were no statistically significant differences among the remaining groups. Rostral cranial fossa percentage values of different breeds in small breed group are presented in Table 4.

**Table 3:** The number of dogs of different breeds in each group, division based on the breed size

Small Breeds		Medium Breeds		Large Breeds		Giant Breeds	
Breed	Number of dogs	Breed	Number of dogs	Breed	Number of dogs	Breed	Number of dogs
Cavalier King Charles Spaniel	1	American Staffordshire Terrier	8	Black Russian Terrier	1	Bernese Mountain Dog	1
Dachshund	3	Australian Shepherd	1	Boxer	3	Great Dane	1
French Bulldog	6	Bavarian Mountain Hound	1	Dobermann Pinscher	1	Italian Mastiff	2
Miniature Pinscher	3	Bull Terrier	1	German Shepherd	9	Newfoundland	1
Pekingese	5	Dalmatian	2	German Wirehaired Pointer	1	Rottweiler	4
Schipperke	1	English Bulldog	2	Labrador Retriever	3		
Scottish Terrier	1	English Beagle	1	Samoyed	1		
Wire Fox Terrier	1	Standard Schnauzer	1	White Swiss Shepherd	1		
Yorkshire Terrier	4

**Table 4:** The rostral cranial fossa percentage in the group of small dogs

Breed	Number of dogs	Value / Range
Pekingese	5	6.293–8.671
French Bulldog	6	8.096–8.985
Yorkshire Terrier	4	7.307–8.670
Cavalier King Charles Spaniel	1	8.803
Miniature Pinscher	3	8.110–9.112
Dachshund	3	7.629–8.864
Schipperke	1	9.455
Scottish Terrier	1	11.114
Wire Fox Terrier	1	10.483

## Discussion

The cranial cavity can be divided into three regions called the RCF, middle cranial fossa and the CCF [15]. CSF, which is produced by the ventricle choroid plexus, flows from the ventricular system into the subarachnoid space via the lateral apertures of the fourth ventricle [15]. This occurs within the CCF, from where CSF is transported along the skull base through the middle cranial fossa to the area of olfactory bulbs located in the RCF [8]. Afterwards, the CSF passes through the cribriform plate foramina into the nasal cavity, where it reaches small lymphatic vessels. This connection has been confirmed in many species using Microfil, the silicon injection rubber compound, which was injected into the cerebellomedullary cistern. Microfil penetrated into the nasal cavity and its small lymphatic vessels [16–18]. Until recently, research on hydrocephalus has focused on the impaired outflow of CSF through the arachnoid villi, which are considered to be the major route for the absorption of CSF from the cranial cavity [19]. A study carried out on sheep suggested that in the case of low intracranial pressure, the majority of the CSF exits the cranial cavity through the cribriform plate and enters the lymphatic vessels of the nasal cavity. The arachnoid villi were described to act as a “safety valve”, which is activated when the intracranial pressure increases [6].

Impaired CSF drainage may result in CNS disorders. A block to CSF flow in the region of the basal cisterns in the middle cranial fossa resulted in hydrocephalus in the rat. Interestingly, the degree of cerebral ventricular enlargement was proportionate to the degree of impaired CSF flow through the cribriform plate [7]. In humans, 8% of surgeries within the skull base result in the formation of postoperative hydrocephalus. This may be a result of disrupted CSF flow caused by the formation of postoperative scars in that region [20]. Hence, hydrocephalus may also develop secondary to a disrupted CSF outflow through the cribriform plate [7].

Hydrocephalus can be congenital or acquired. Toy and brachycephalic breeds, such as the Pekingese, Yorkshire Terrier, Maltese, Miniature Poodle, Pomeranian, Chihuahua, Pug, Boston Terrier and English Bulldog are predisposed to congenital hydrocephalus [2, 3]. This type of hydrocephalus is most frequently caused by a stenosis of the mesencephalic aqueduct although the exact obstruction site and the precise cause of the hydrocephalus often remains undiagnosed [2, 21]. Therefore, impaired flow of the CSF out of the cranial cavity may be a cause of congenital hydrocephalus. We found that the rostral cranial fossa percentage was smaller in small breed dogs compared to the other groups of dogs. Similar findings were obtained when comparing brachycephalic dogs with mesati- and dolichocephalic dogs. In the group of small breed dogs, the Yorkshire terrier, Pekingese, French bulldog, Miniature Poodle, Miniature Pinscher and Dachshund were found to have a decreased volume of the RCF. In the group of brachycephalic dogs, the size of the RCF was decreased in the French Bulldog, English Bulldog and Pekingese. Some of those breeds are predisposed to congenital hydrocephalus [3]. Ventricular enlargement without neurological symptoms has been described in the Yorkshire terrier and English bulldog. [22, 23]. Our data indicate that the RCF volume was decreased in some breeds predisposed to congenital hydrocephalus. However, the reduction of the RCF volume does not seem to be the sole cause of the disruption of intracranial CSF circulation. This is because the prevalence of hydrocephalus in breeds such as the Dachshund and Miniature Pinscher, which also have a reduced RCF volume, is much smaller. In theory, hydrocephalus in predisposed small and brachycephalic breeds may be caused by the overcrowding of brain structures in the RCF, impeding CSF outflow via the cribriform plate. However, further studies are needed to clarify this. The percentage volume of the brain parenchyma in the RCF in breeds with different morphotypes and size should be studied further. That would prove whether brain structures are crowded in the region of the cribriform plate.

The concept of the CSF flow being disrupted by the crowded brain structures is not novel. CCF pathologies may also cause disturbances in CSF flow. Some breeds of dogs suffer from CM [10, 12, 24, 25], which is caused by occipital bone hypoplasia leading to a decreased CCF volume. This leads to “overcrowding” of brain structures within the CCF and causes an obstruction of CSF flow at the foramen magnum, which is considered one of the causes of SM. However, the pathophysiology of SM may be multifactorial [26, 27]. CM and SM most often occur in the Cavalier King Charles Spaniel [28]. They have also been described in the Griffon Bruxellois, Yorkshire Terrier, Maltese and the Poodle [12, 25, 29]. Other breeds of dogs that suffer from CM, such as the Chihuahua, Boston Terrier and Papillion, are at risk of developing SM [30]. The majority of those breeds are brachycephalic, toy or both. In some cases, SM is accompanied by ventricular enlargement [25, 29]. This is in accordance with our findings, where we observed a decreased volume of the RCF, a site of CSF outflow, in brachycephalic and small breed dogs. In breeds with CM, the volume of the CCF and RCF may be decreased. That may significantly disrupt CSF circulation. The association between a decreased RCF volume and SM warrants further study.

## Conclusions

The determination of volume ratios between different compartments of the cranial cavity is essential to understand certain CNS disorders associated with disrupted CSF circulation. Our results indicate that the ratio of RCF volume to CC volume is lower in small and brachycephalic breeds of dogs. A small rostral cranial fossa percentage is found in dogs predisposed to congenital hydrocephalus. We are aware that our findings do not prove impaired CSF outflow through the cribriform plate. However, they could form the basis for future research on this area of the cranial cavity. The ratios of the brain parenchyma within the RCF to the volume of the RCF should be calculated. The flow of CSF through the cribriform plate to the NL should be thoroughly studied in dogs with hydrocephalus.

## Methods

### Material

Seventy-one skulls obtained from adult dogs of both genders (35 males and 36 females) from 2 to 16 years old were analyzed. Mixed-breed dogs and mongrel dogs were not included in the study. The skulls were divided based on the breed size into small, medium, large and giant breed group. Based on the skull index individuals were divided into the dolichocephalic, mesaticephalic 1, mesaticephalic 2 and brachycephalic breed group [15].

The skulls were obtained from animals euthanised by veterinary doctors at the Small Animal Clinic of the Department of Clinical Sciences, Faculty of Veterinary Medicine, of the Warsaw University of Life Sciences. The animals were not euthanized for the purpose of this study. The owners consented to the euthanasia and to the use of the animal tissue for scientific purposes. None of the animals had any neurological symptoms prior to euthanasia. Examined animals were adults, with closed skull sutures and without dome-shaped skull. According to the Polish law, the post mortem use of tissue does not require the approval from the Ethics Committee (Parliament of the Republic of Poland, 2015). The corpses were subjected to a process of maceration. Soft tissue were removed from cranial cavity prior to CT examination.

### CT analysis

**Fig. 1:**
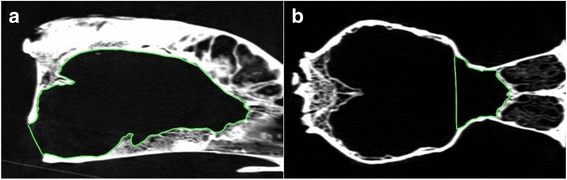
Regions of interest used to calculate volCC (**a**) and volRCF (**b**)

**Fig. 2:**
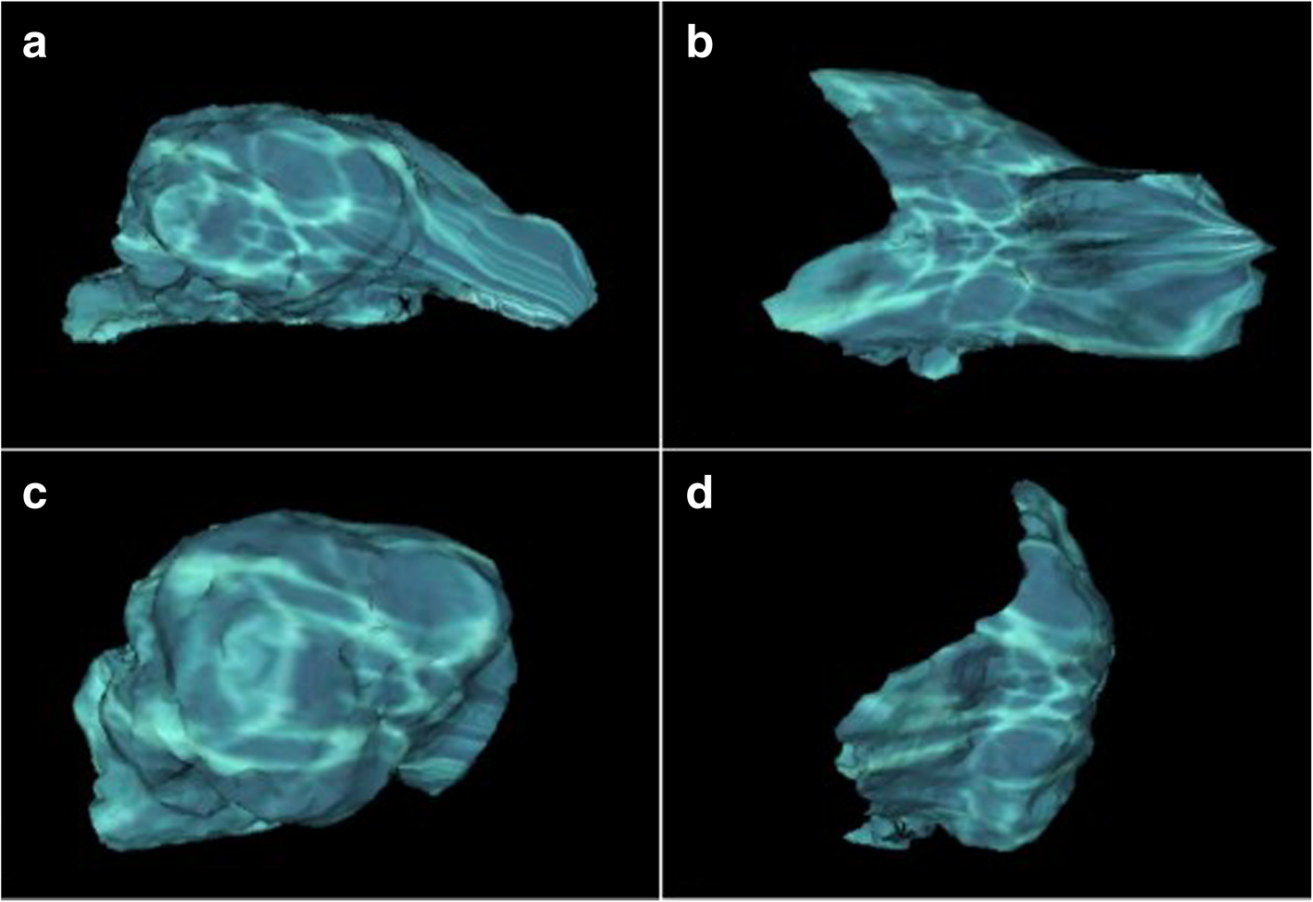
The three-dimensional model of cranial cavity (**a**) and rostral cranial fossa (**b**) in dolichocephalic dog; cranial cavity (**c**) and rostral cranial fossa (**d**) in brachycephalic dog

Computed tomography images of the skulls placed in ventral recumbency were acquired with a helical CT scanner (Siemens SOMATOM Emotion 16 CT Scanner) and a slice thickness of 0.75 mm to provide detailed image of the cranial cavity. Images were reconstructed with bone filter and displayed using a bone window (window width 2000 HU and window level 350 HU). In some cases the window was adjusted manually to better outline specific bony structures. CT data were transferred as a DICOM file to an image analysis workstation with Osirix® software in order to perform the image analysis. The originally obtained transverse slices were reformatted into the sagittal and dorsal planes. The volCC (volume of cranial cavity) and volRCF (volume of the rostral cranial fossa) were calculated. The volCC was obtained using the slice by slice method. The region of interest (ROI) was selected on each slice in the sagittal plane (Fig. 1). Next, a three-dimensional model presenting the volume was created using the ROI Volume function (Fig. 2). The dorsal and ventral margin of the foramen magnum formed the caudal border of the cranial cavity. The volRCF was obtained through the same method (Figs. 1 and 2). The ROI was selected using the dorsal plane. The bone protrusion corresponding to the presylvian fissure formed the caudal margin of this ROI. Only those skulls, where this bony protrusion was easily identifiable, were included in the study. At the skull base, the caudal edge of the prechiasmatic sulcus formed the caudal margin of the RCF. Those values were used to calculate the rostral cranial fossa percentage = volRCF/volCC × 100.

The linear AP, which is the distance between the Akrokranion – Prosthion, was measured in the median plane. The Akrokranion is the most caudal point of the calvaria and the Prosthion is the most rostral point of the interincisive suture. The linear ZyZy, which is the distance between the most protruding points of the zygomatic arch, was measured in the dorsal plane. Those measurements were used to calculate the skull index = ZyZy/AP × 100 [15].

### Statistical analysis

The Statistica 10.0 (StatSoft, Inc.) software was used to assess the results. The distribution of the variables in the examined population was evaluated using the W Shapiro-Wilk test. The obtained data had a normal distribution, and they were analysed using the one-way analysis of variance. The effect of the breed size and the morphotype of the animals on the rostral cranial fossa percentage were assessed separately. Statistical significance between the groups was calculated using Tukey’s honestly significant difference (HSD) test.

## Additional file

Additional file 1: Individuals value of AP, ZyZy, volRCF, volCC and volRCF/volCC ratio (RCF/CC). (XLS 20 kb)

## Abbreviations

AP: Distance between the Akrokranion – Prosthion
CC: Cranial cavity
CCF: Caudal cranial fossa
CM: Chiari-like malformation
CSF: Cerebrospinal fluid
NL: Nasal lymphatics
RCF: Rostral cranial fossa
ROI: Region of interest
volCC: Volume of cranial cavity
volRCF: Volume of the rostral cranial fossa
ZyZy: Distance between the most protruding points of the zygomatic arch
